# Inhibition of interleukin-1β-induced endothelial tissue factor expression by the synthetic cannabinoid WIN 55,212-2

**DOI:** 10.18632/oncotarget.11367

**Published:** 2016-08-18

**Authors:** Antje Scholl, Igor Ivanov, Burkhard Hinz

**Affiliations:** ^1^ Institute of Toxicology and Pharmacology, Rostock University Medical Center, D-18057 Rostock, Germany

**Keywords:** WIN 55, 212-2, tissue factor, endothelial cells, ceramide, MAP kinases

## Abstract

The role of cannabinoids in thrombosis remains controversial. In view of the primary importance of tissue factor (TF) in blood coagulation and its involvement in the pathology of several cardiovascular, inflammatory and neoplastic diseases, a regulation of this initial procoagulant signal seems to be of particular interest. Using human umbilical vein endothelial cells (HUVEC) the present study investigated the impact of the synthetic cannabinoid WIN 55,212-2 on interleukin (IL)-1β-induced TF expression and activity. WIN 55,212-2 caused a time- and concentration-dependent suppression of IL-1β-induced TF protein accompanied by decreases in TF mRNA and activity. Inhibition of TF protein expression by WIN 55,212-2 was mimicked by its cannabinoid receptor-inactive enantiomer WIN 55,212-3 but not by structurally unrelated phyto-, endo- and synthetic cannabinoids. In addition, the inhibitory effect of WIN 55,212-2 was not reversed by antagonists to cannabinoid receptors (CB_1_, CB_2_) or transient receptor potential vanilloid 1. Mechanistic approaches revealed WIN 55,212-2 to suppress IL-1β-induced TF expression via inhibition of ceramide formation and via decreased phosphorylation of p38 mitogen-activated protein kinase (MAPK) and c-Jun N-terminal kinases. Further inhibitor experiments demonstrated neutral sphingomyelinase (nSMase) to confer ceramide generation upon IL-1β treatment with the parallel IL-1β-mediated activation of MAPKs occurring via an nSMase-independent pathway. Finally, a receptor-independent inhibition of IL-1β-induced TF protein by WIN 55,212-2 was confirmed in human blood monocytes. Collectively, this data provide a hitherto unknown receptor-independent anticoagulatory action of the cannabinoid WIN 55,212-2.

## INTRODUCTION

Tissue factor (TF), formerly known as thromboplastin, is a critical initiator of physiologic and pathophysiologic coagulation. The 47-kDa transmembrane glycoprotein binds factor VIIa to form a complex that cleaves factor IX and factor X, ultimately leading to fibrin formation (for review see [1, 2]). The expression of TF is regulated predominantly at the transcriptional level. TF is constitutively expressed in vascular smooth muscle cells leading to its rapid exposure to circulating blood following disruption of the endothelial layer [3]. In endothelial cells and monocytes TF is expressed to a limited extent only, but becomes upregulated by diverse stimuli, such as lipopolysaccharide, proinflammatory cytokines (e.g. tumor necrosis factor (TNF) and interleukin (IL)-1β), growth factors, oxidized low density lipoprotein, hypoxia, shear stress and oxidants (for review see [1, 2]). Furthermore, emerging evidence suggests that the expression of TF on endothelial cells of pathologic blood vessels is associated with solid tumors [4–9]. By stimulating TF expression and inhibiting the thrombomodulin/protein C anticoagulation pathway, proinflammatory cytokines, including TNF and IL-1β, may trigger endothelial cells into a procoagulant, clot-promoting state. In addition, a cytokine-driven increased TF expression on the monocyte surface facilitates the interaction of the monocyte with activated platelets and endothelial cells via binding of P-selectin, resulting in fibrin deposition and thrombus formation (for review see [10, 11]).

TF-dependent coagulation has been implicated to promote thrombotic episodes in a variety of clinical disorders, including cardiovascular diseases, septic shock and cancer. Accordingly, TF expression has been identified in all stages of atherosclerotic lesions and significantly higher levels of circulating soluble TF have been found in patients with acute myocardial infarction and unstable angina (for review see [12]). Clinically, inhibitors of cytokine action have been demonstrated to regulate the prothrombotic actions of TF in sepsis. For example, administration of recombinant IL-1 receptor antagonist, the naturally occurring inhibitor of IL-1, has been shown to decrease coagulation in patients with sepsis and in septic baboons [13, 14].

TF-dependent signaling has been also implicated in tumor growth, angiogenesis, metastasis and thrombosis in patients with cancer (for review see [15]). As a matter of fact, upregulation of TF by cancer as well as by certain host cells influences tumor progression via multiple mechanisms (for review see [16]). Collectively, this data strongly imply inhibition of TF action as an attractive pharmacotherapeutic target.

Despite increasing knowledge on the therapeutic benefits of cannabinoids in diverse medical fields, the impact of these substances on hemostasis and thrombosis remains controversial (for review see [17]). Whereas acute administration of cannabis has been identified as a risk factor for initiating a myocardial infarction [18], long-term cannabis smoking was not associated with increased cardiovascular risk [19]. In line with a thrombogenic action of cannabis, Δ^9^-tetrahydrocannabinol (THC), the main psychoactive ingredient of marijuana, was previously shown to increase the expression of glycoprotein IIb-IIIa and P-selectin on platelets [20]. Likewise, the endocannabinoids anandamide (N-arachidonoylethanolamine, AEA) and 2-arachidonoylglycerol (2-AG) have been demonstrated to activate platelets when tested at micromolar concentrations [21–23]. On the other hand, metabolism of endocannabinoids by the endothelial prostacyclin synthase has been shown to interfere with the expression of TF [24].

The present study focused on the impact of the synthetic cannabinoid WIN 55,212-2 on the expression and activity of IL-1β-induced TF in human umbilical vein endothelial cells (HUVEC). The aminoalkylindole derivative is a full agonist at the CB_1_ cannabinoid receptor and has much higher affinity than THC for this receptor [25]. In contrast to other cannabinoids (e.g. AEA) which activate transient receptor potential vanilloid 1 (TRPV1), WIN 55,212-2 inhibits TRPV1 functional activities [26] and modulates TRPV1 activation by altering receptor phosphorylation [27]. WIN 55,212-2 was chosen for more detailed analysis on the basis of several studies showing an interference of this cannabinoid with diverse pathways elicited by proinflammatory cytokines [28–35] via mechanisms involving both cannabinoid receptor-dependent [28, 30, 33] and -independent actions [29, 32]. Here we provide first-time proof for WIN 55,212-2 to decrease IL-1β-induced expression and activity of TF in endothelial cells. The underlying receptor-independent pathway was shown to involve an inhibition of neutral sphingomyelinase (nSMase)-dependent ceramide formation as well as an interference with the activation of p38 mitogen-activated protein kinase (MAPK) and c-Jun N-terminal kinases (JNK).

## RESULTS

### Time course and concentration dependence of the inhibitory effect of WIN 55,212-2 on IL-1β-induced TF expression

**Figure 1:**
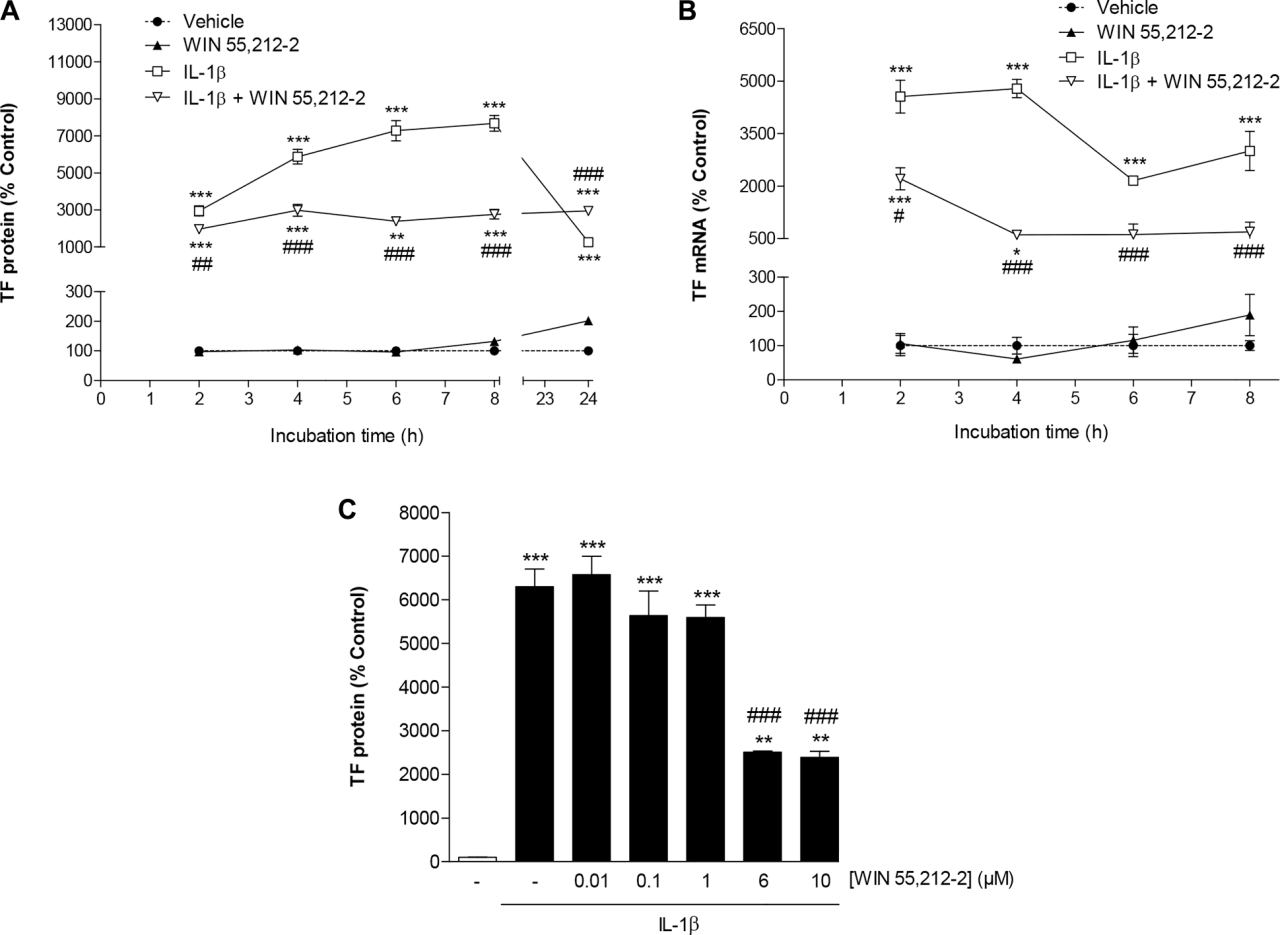
Impact of WIN 55,212-2 on TF expression in HUVEC Time course of TF protein (**A**) and mRNA (**B**) expression following incubation of cells with WIN 55,212-2 in the presence or absence of IL-1β. Concentration-dependent effect of WIN 55,212-2 on IL-1β-induced TF protein levels (C). Cells were incubated with WIN 55,212-2 at 10 μM (A, B) or at the indicated concentrations (**C**) for the indicated times (A, B) or for 8 h (C) in the presence or absence of IL-1β (10 ng/ml). Percent control represents comparison with vehicle-treated cells (100%) in the absence of test substance. Values are means + SEM of *n* = 3 (A, C) or *n* = 3–4 (B) per group. *P < 0.05, **P < 0.01, ***P < 0.001 vs. vehicle control; ^#^*P* < 0.05, ^##^*P* < 0.01, ^###^*P* < 0.001 vs. IL-1β-treated cells, ANOVA plus post hoc Bonferroni test.

Incubation of HUVEC for 2 to 24 h with IL-1β led to a continuous increase of TF protein during the first 8 h up to a 76.8-fold induction over vehicle (Figure 1A). Induction of TF mRNA by IL-1β became evident within 2 h after stimulation and remained significantly increased up to 8 h after stimulation (Figure 1B). Treatment of HUVEC with the synthetic cannabinoid WIN 55,212-2 at 10 μM concomitantly to IL-1β was associated with a significant suppression of IL-1β-induced TF protein (Figure 1A) and mRNA levels (Figure 1B) within an 8-h incubation period.

To investigate a possible concentration dependence of WIN 55,212-2, different concentrations of this cannabinoid were tested for its impact on IL-1β-induced TF protein expression using an 8-h incubation period. According to Figure 1C, a significant inhibition of IL-1β-induced TF expression was observed at a threshold concentration of 6 μM WIN 55,212-2. Without concomitant incubation with IL-1β, an increase of basal TF protein levels was not elicited with concentrations of WIN 55,212-2 up to 6 μM (data not shown). In case of the 10-μM concentration, a significant upregulation of TF was only obtained when summarizing several experiments (vehicle, 100% ± 1%; WIN 55,212-2 (10 μM), 145% ± 12%; means ± SEM of *n* = 15 per group, *P* < 0.01, Student's unpaired *t* test). However, in view of the variable effect on basal TF expression, further investigations of the underlying mechanism were not undertaken.

### Impact of WIN 55,212-2 on IL-1β-induced TF activity and IL-1β-mediated decrease of aPTT

**Figure 2:**
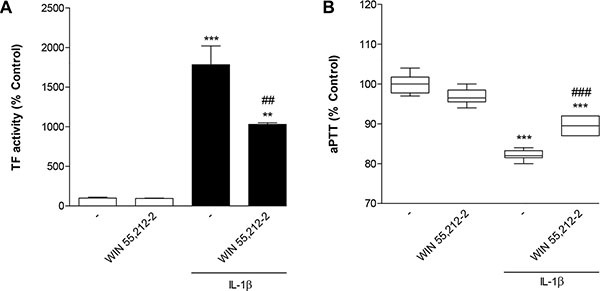
Impact of WIN 55,212-2 on IL-1β-induced TF activity (A) and IL-1β-mediated decrease of aPTT (B) HUVEC were incubated with WIN 55,212-2 at 10 μM for 8 h in the presence or absence of IL-1β (10 ng/ml). Percent control represents comparison with vehicle-treated cells (100%) in the absence of test substance. Values (A) are means + SEM of *n* = 3 per group. In the box plot (B, *n* = 6 per group), boxes extend from the 25th percentile to the 75th percentile, with a horizontal line inside the box at the median. Whiskers indicate minimum and maximum values, respectively. **P < 0.01, ***P < 0.001 vs. vehicle control; ^##^*P* < 0.01, ^###^*P* < 0.001 vs. IL-1β-treated cells, ANOVA plus post hoc Bonferroni test.

To assess the functional relevance of the observed regulations of TF expression by WIN 55,212-2 and IL-1β, its impact on TF activity and activated partial thromboplastin time (aPTT) was investigated next. According to Figure 2A IL-1β caused a 17.8-fold upregulation of TF activity that was significantly attenuated in the presence of WIN 55,212-2. Furthermore, treatment of cells with IL-1β was associated with a significant decrease of aPTT that was again partially reversed by WIN 55,212-2 (Figure 2B).

### Evaluation of the involvement of cannabinoid-activated receptors in TF inhibition by WIN 55,212-2

**Figure 3:**
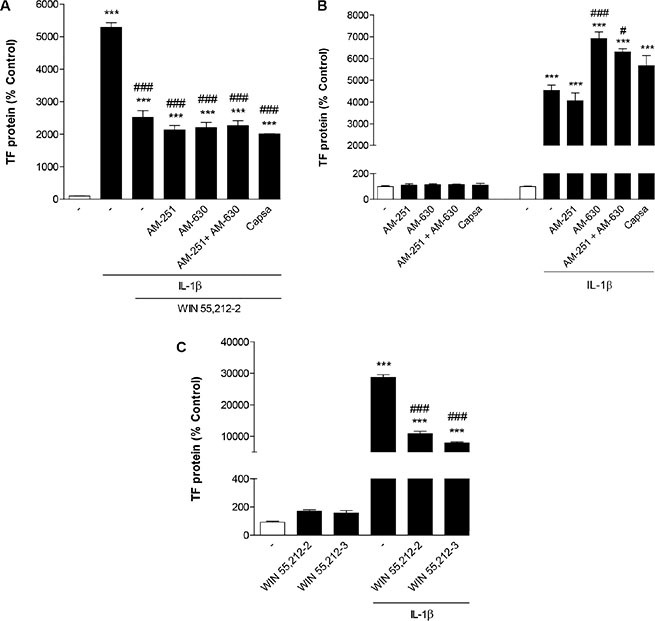
Involvement of cannabinoid-activated receptors in inhibition of IL-1β-induced TF protein expression by WIN 55,212-2 Effect of AM-251 (CB_1_ antagonist), AM-630 (CB_2_ antagonist) and capsazepine (Capsa, TRPV1 antagonist) on the WIN 55,212-2-mediated inhibition of IL-1β-induced TF protein expression (**A**) or basal and IL-1β-induced TF protein levels (**B**). Cells were pretreated with the respective receptor antagonist (all tested at a final concentration of 1 μM) for 1 h. IL-1β (10 ng/ml), WIN 55,212 2 (10 μM) or vehicle were added subsequently and the incubation was continued for 8 h. (**C**) Effect of the cannabinoid receptor-inactive enantiomer WIN 55,212-3 on basal and IL-1β-induced TF protein expression. Cells were incubated with equimolar concentrations of WIN 55,212-3 and WIN 55,212-2 (both at 10 μM) for 8 h. Percent control represents comparison with vehicle-treated cells (100%) in the absence of test substance. Values are means + SEM of *n* = 3 per group. ***P < 0.001 vs. corresponding vehicle control; ^#^*P* < 0.05, ^###^*P* < 0.001 vs. IL-1β, ANOVA plus post hoc Bonferroni test.

To ascertain a possible role of CB receptors and TRPV1 in the inhibitory action of WIN 55,212-2 on IL-1β-induced TF protein expression, cells were preincubated with the CB_1_ receptor antagonist AM-251, the CB_2_ receptor antagonist AM-630 or the TRPV1 antagonist capsazepine. All antagonists were used at a concentration of 1 μM, which has been reported to be within the range of concentrations inhibiting CB_1_-, CB_2_- and TRPV1-dependent events [36–40]. However, none of the three substances tested alone or in combination reversed the inhibitory action of WIN 55,212-2 on IL-1β-induced TF expression (Figure 3A). Treatment of cells with the antagonists alone without IL-1β and WIN 55,212-2 caused no significant change of basal TF expression in HUVEC (Figure 3B). In the presence of IL-1β, however, additional treatment with AM-630 as well as the combination of AM-251 and AM-630 was associated with a further significant increase of IL-1β-induced TF protein levels (Figure 3B).

In additional experiments the cannabinoid receptor-inactive enantiomer WIN 55,212-3 which shares the aminoalkylindole structure with WIN 55,212-2 was tested for its impact on IL-1β-induced TF expression. As shown in Figure 3C, WIN 55,212-3 mimicked the action of WIN 55,212-2 leading to a likewise pronounced inhibitory effect on TF increase by IL-1β.

The possibility that decreased availability of TF levels by WIN 55,212-2 and WIN 55,212-3 in IL-1β-treated HUVEC was an unspecific cytotoxicity-related phenomenon can be ruled out in view of the fact that all TF protein values measured had been normalized to cellular protein. Nonetheless, cellular viability was measured under experimental conditions similar to those used for determination of TF protein. The results of these experiments indicate that incubation with WIN 55,212-2 impaired basal viability, whereas treatment of cells with the somewhat more effective inhibitor of TF expression, WIN 55,212-3, did not result in a significant change of viability. Viability rates measured after an 8-h treatment with the respective substances were as follows: vehicle, 100% ± 5%; WIN 55,212-2 (10 μM), 59% ± 7% ***; WIN 55,212-3 (10 μM), 110% ± 6%; IL-1β, 70% ± 5% **; IL-1β + WIN 55,212-2 (10 μM), 64% ± 5% ***; IL-1β + WIN 55,212-3 (10 μM), 67% ± 3% ***, means ± SEM of n = 8 per group, **P < 0.01, ***P < 0.001 vs. vehicle, one-way ANOVA plus post hoc Bonferroni test.

### Impact of cannabinoids structurally unrelated to WIN 55,212-2 on basal and IL-1β-induced TF expression

To determine whether the inhibitory effect on IL-1β-induced TF expression was unique for WIN 55,212-2 or shared by structurally unrelated cannabinoids, additional experiments were performed with diverse endo-, phyto- and synthetic cannabinoids.

**Figure 4:**
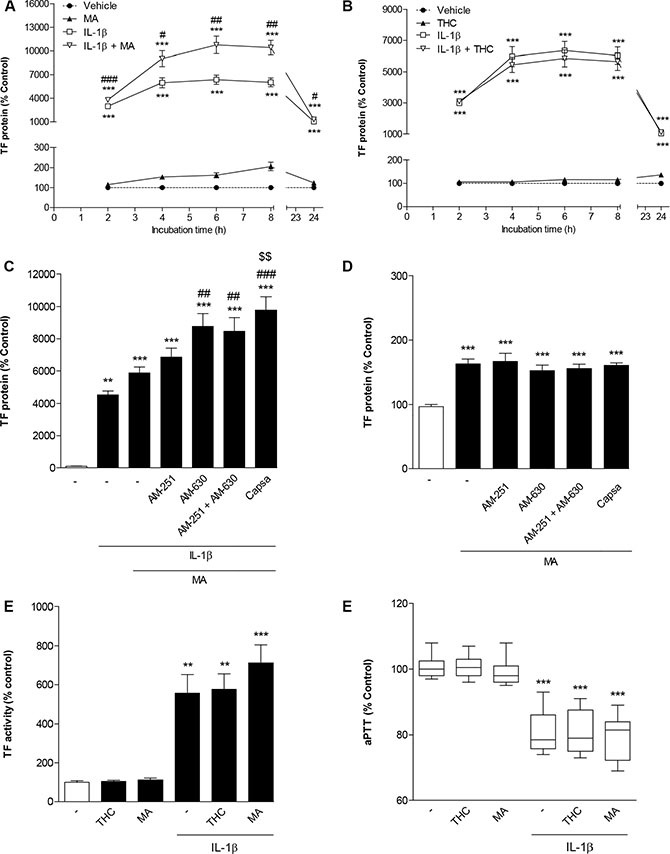
Impact of R(+)-methanandamide (MA) and Δ^9^-tetrahydrocannabinol (THC) on basal and IL-1β-induced TF expression Time dependencies of TF protein expression following incubation of cells with MA (**A**) or THC (**B**) in the presence or absence of IL-1β. Effect of AM-251 (CB_1_ antagonist), AM-630 (CB_2_ antagonist) and capsazepine (Capsa, TRPV1 antagonist) on the MA-mediated increase of IL-1β-induced (**C**) or basal TF protein levels (**D**). Impact of MA and THC on IL-1β-induced TF activity (**E**) and on IL-1β-mediated decrease of aPTT (**F**). Cells were incubated with MA at 10 μM or THC at 3 μM for the indicated times (A, B) or for 8 h (C–F) in the presence or absence of IL-1β (10 ng/ml). Receptor antagonists (C, D) were added to cultures 1 h prior to MA. Percent control represents comparison with vehicle-treated cells (100%) in the absence of test substance. Values are means + SEM of *n* = 3 (A, B, C, E) or *n* = 6 (D) per group. In the box plot (F, *n* = 17–18 per group), boxes extend from the 25th percentile to the 75th percentile, with a horizontal line inside the box at the median. Whiskers indicate minimum and maximum values, respectively. **P < 0.01, ***P < 0.001 vs. corresponding vehicle control; ^#^*P* < 0.05, ^##^*P* < 0.01, ^###^*P* < 0.001 vs. IL-1β, ^§§^*P* < 0.01 vs. IL-1β + MA, ANOVA plus post Bonferroni test.

In a first set of experiments this investigation was focused on R(+)-methanandamide (MA), a hydrolysis-stable analogue of the endocannabinoid AEA, and THC, the major psychoactive cannabinoid of the cannabis plant. As shown in Figure 4A and 4B, time course experiments revealed no decrease of IL-1β-induced TF protein in the presence of both cannabinoids. In case of MA, IL-1β-induced TF levels were even significantly enhanced (Figure 4A). Albeit to a lesser extent, MA was likewise found to enhance basal TF levels (Figure 4A). However, in both cases no clear concentration dependence was observed with significant upregulations being confined to 10-μM concentrations of MA (Table 1). Subsequent experiments addressing the role of cannabinoid-activated receptors in these responses revealed no inhibition of MA-mediated increases of both IL-1β-induced (Figure 4C) and basal TF protein levels (Figure 4D) in the presence of antagonists to CB_1_, CB_2_ and TRPV1, respectively. In addition, potentiation of IL-1β-induced TF expression by MA was not inhibited by the cyclooxygenase (COX) inhibitor indometacin (data not shown), thus excluding prostanoids generated upon COX-dependent metabolization of MA or its hydrolysis product arachidonic acid to confer the observed effect. On the functional level, both MA and THC did not lead to a significant increase of IL-1β-induced TF activity (Figure 4E). Moreover, both cannabinoids left the IL-1β-caused decrease of aPTT virtually unaltered (Figure 4F).

**Table 1:** Concentration-dependent effect of R(+)-methanandamide (MA) on basal and IL-1β-induced TF protein levels

Treatment	TF protein (% control)
Vehicle	100 ± 5
MA (0.01 μM)	101 ± 5
MA (0.1 μM)	95 ± 4
MA (1 μM)	93 ± 7
MA (3 μM)	106 ± 7
MA (10 μM)	136 ± 9 **
Vehicle	100 ± 5
IL-1β	7541 ± 541 ***
IL-1β + MA (0.01 μM)	7213 ± 245 ***
IL-1β + MA (0.1 μM)	7600 ± 673 ***
IL-1β + MA (1 μM)	8221 ± 541 ***
IL-1β + MA (3 μM)	8483 ± 578 ***
IL-1β + MA (10 μM)	11264 ± 128 *** ###

Cells were incubated with MA for 8 h in the presence or absence of IL-1β. Percent control represents comparison with vehicle-treated cells (100%) in the absence of test substance. Values are means + SEM of *n* = 3 per group.

** *P* < 0.01

*** *P* < 0.001 vs. corresponding vehicle control

### *P* < 0.001 vs. IL-1β, ANOVA plus post hoc Dunnett (upper part) or Bonferroni test (lower part).

Additional experiments yielded significant increases in basal and IL-1β-induced TF levels by the endocannabinoid AEA, whereas the endocannabinoid 2-AG was virtually inactive in this respect (Table 2). In addition, basal TF expression was significantly elevated by arachidonic acid, the hydrolysis product of AEA, and by the phytocannabinoid cannabidiol (Table 2). A significant increase of IL-1β-stimulated TF expression was also observed after incubation of cells with the CB_2_ agonist JWH-133 (Table 2).

**Table 2:** Impact of the endocannabinoids anandamide (AEA, 10 μM) and 2-arachidonoylglycerol (2-AG, 10 μM), the phytocannabinoid cannabidiol (CBD, 6 μM), the synthetic cannabinoid JWH-133 (10 μM) as well as the endocannabinoid hydrolysis product arachidonic acid (10 μM) on basal and IL-1β-induced TF expression

Treatment	TF protein (% control)
Vehicle	100
AEA (10 μM)	316 ± 18 ***
2-AG (10 μM)	95 ± 8
Arachidonic acid (10 μM)	167 ± 11 *
Cannabidiol (6 μM)	279 ± 27 ***
JWH-133 (10 μM)	161 ± 4
Vehicle	100 ± 3
IL-1β	8322 ± 190 ***
IL-1β + AEA (10 μM)	12688 ± 555 *** ###
IL-1β + 2-AG (10 μM)	8683 ± 363 ***
IL-1β + Arachidonic acid (10 μM)	8711 ± 507 ***
Vehicle	100 ± 4
IL-1β	8184 ± 541 ***
IL-1β + Cannabidiol (6 μM)	9556 ± 448 ***
IL-1β + JWH-133 (10 μM)	10293 ± 150 *** ##

Cells were incubated with the respective compound for 8 h in the presence or absence of IL-1β. Percent control represents comparison with vehicle-treated cells (100%) in the absence of test substance. Values are means + SEM of *n* = 3 per group.

* *P* < 0.05

*** *P* < 0.001 vs. corresponding vehicle control;

## *P* < 0.01

### *P* < 0.001 vs. IL-1β, ANOVA plus post hoc Dunnett (upper part) or Bonferroni (lower two parts) test. With respect to different vehicle controls in the first set of data that were considered for statistical evaluation of the respective experiment, an SEM of the vehicle control is not given in this case.

### Role of ceramide in IL-1β-induced TF expression and its modulation by WIN 55,212-2

Looking for second messengers conferring the inhibitory action of WIN 55,212-2 on TF expression, different potential pathways underlying IL-1β-induced TF expression were analyzed. In a first approach, the role of ceramide in IL-1β-induced TF expression and its potential modulation by WIN 55,212-2 was investigated.

**Figure 5:**
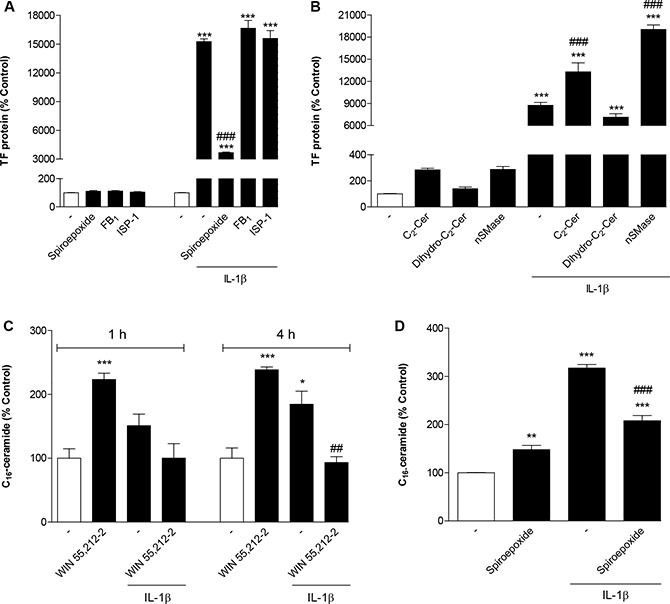
Role of ceramide in IL-1β-induced TF expression and its modulation by WIN 55,212-2 (**A**) Effect of nSMase spiroepoxide inhibitor (selective inhibitor of nSMase; 10 μM), fumonisin B_1_ (selective inhibitor of ceramide synthase; 50 μM) and ISP-1 (serine palmitoyltransferase inhibitor; 10 μM) on basal and IL-1β-induced TF protein levels. Cells were pretreated with the respective inhibitor for 1 h (fumonisin B_1_, ISP) or 1.5 h (nSMase spiroepoxide inhibitor). IL-1β or vehicle were added subsequently and the incubation was continued for 8 h. (**B**) Effect of C_2_-ceramide (10 μM), dihydro-C_2_-ceramide (10 μM) and nSMase from *B. cereus* (10 mU/ml) on basal and IL-1β-induced TF protein levels. Cells were incubated with the respective substance in the presence or absence of IL-1β for 8 h. (**C**, **D**) Impact of WIN 55,212-2 (C, 10 μM) and nSMase spiroepoxide inhibitor (D, 10 μM) on basal and IL-1β-induced cellular concentrations of C_16_-ceramide. Cells were treated with IL-1β, WIN 55,212-2 or vehicle for the times indicated (C). In case of (D) cells were pretreated with nSMase spiroepoxide inhibitor for 1.5 h. IL-1β or vehicle were added subsequently and the incubation was continued for 4 h. Percent control represents comparison with vehicle-treated cells (100%) in the absence of test substance. Values are means + SEM of *n* = 3 per group. *P < 0.05, **P < 0.01, ***P < 0.001 vs. corresponding vehicle control; ^##^*P* < 0.01, ^###^*P* < 0.001 vs. IL-1β, ANOVA plus post hoc Bonferroni test.

In initial experiments the ceramide-generating enzymes nSMase and ceramide synthase were focused on as possible targets of IL-1β. The question of whether IL-1β acted through nSMase to induce TF expression was examined by use of a selective inhibitor of nSMase, referred to as nSMase spiroepoxide inhibitor [41]. Experiments performed to assess the impact of de novo ceramide synthesis were performed by use of the potent and selective inhibitor of ceramide synthase fumonisin B_1_ [42] as well as ISP-1, an inhibitor of serine palmitoyltransferase. Whereas treatment of cells with nSMase spiroepoxide inhibitor was associated with a substantial suppression of IL-1β-induced TF formation, fumonisin B_1_ and ISP-1 failed to elicit such effect (Figure 5A). All inhibitors did not alter basal TF protein levels (Figure 5A).

To further confirm whether ceramide confers induction of TF expression, cells were incubated with C_2_-ceramide, a cell-permeable, short-chain ceramide analog. As negative control cells were stimulated with dihydro-C_2_-ceramide, an inactive analog of C_2_-ceramide. As shown in Figure 5B, C_2_-ceramide increased basal TF protein levels and significantly potentiated IL-1β-induced TF concentrations in an overadditive manner, whereas dihydro-C_2_-ceramide was inactive in both respects. In addition, the stimulatory effect of ceramide on basal and IL-1β-induced TF protein formation was confirmed by incubating cells with nSMase from *B. cereus* (Figure 5B).

In order to prove the generation of ceramide upon treatment of HUVEC with IL-1β and a possible interference by WIN 55,212-2, cell lysates were assayed for C_16_-ceramide following different incubation times with test substances. As demonstrated in Figure 5C, a significant induction of cellular ceramide levels by IL-1β became significant after a 4-h incubation yielding a 1.85-fold increase over basal. This increase was fully reversed in the presence of WIN 55,212-2 (Figure 5C). Interestingly, incubation of cells with WIN 55,212-2 alone was also associated with an induction of cellular ceramide to an extent comparable to that elicited by IL-1β (Figure 5C). The involvement of nSMase in conferring cellular increases of ceramide elicited by IL-1β was confirmed by use of the nSMase spiroepoxide inhibitor that antagonized the action of IL-1β (Figure 5D).

### Role of MAPKs in IL-1β-induced TF expression and its modulation by WIN 55,212-2

**Figure 6:**
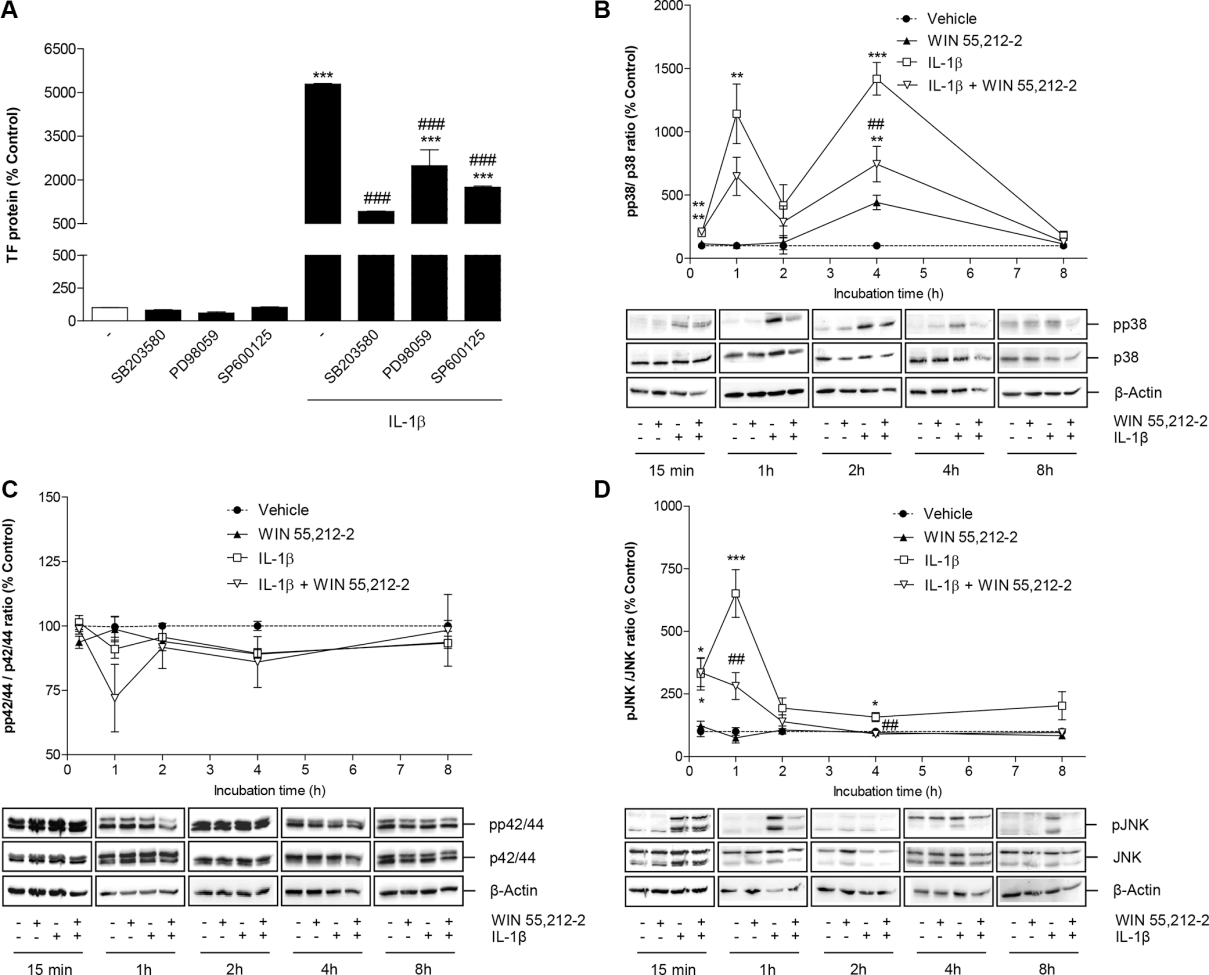
Role of MAPKs in IL-1β-induced TF expression and its mo dulation by **WIN 55,212-2.** (**A**) Effect of SB203580 (inhibitor of p38 MAPK; 10 μM), PD98059 (inhibitor of p42/44 MAPK activation; 10 μM) and SP600125 (inhibitor of JNK; 10 μM) on basal and IL-1β-induced TF protein levels. Cells were pretreated with the respective inhibitor for 1 h. IL-1β, WIN 55,212-2 or vehicle were added subsequently and the incubation was continued for 8 h. (**B**–**D**) Impact of WIN 55,212-2 on the activation of p38 MAPK (B), p42/44 MAPK (C) and JNK (D) under basal and IL-1β-modulated conditions. Cells were incubated with WIN 55,212-2 at 10 μM for the indicated times in the presence or absence of IL-1β. Percent control (A–D) represents comparison with vehicle-treated cells (100%) in the absence of test substance. Values shown in B–D represent densitometric analyses of blots with the phosphorylated forms being normalized to the respective unphosphorylated form. ß-actin is shown as loading control. All values are means + SEM of *n* = 3 per group. *P < 0.05, **P < 0.01, ***P < 0.001 vs. corresponding vehicle control; ^##^*P* < 0.01, ^###^*P* < 0.001 vs. IL-1β, ANOVA plus post hoc Bonferroni test.

To investigate whether IL-1β-induced TF formation in HUVEC was a downstream event of p38 MAPK, p42/44 MAPK or JNK activation, cells were incubated with IL-1β in the presence of SB203580, an inhibitor of p38 MAPK, PD98059, an inhibitor of p42/44 MAPK activation, and SP600125, an inhibitor of JNK. According to Figure 6A, inhibition of all kinases was associated with a partial but significant inhibition of IL-1β-induced TF formation with the most pronounced effect observed with the p38 MAPK inhibitor.

Time course experiments performed over an 8-h incubation period revealed an IL-1β-induced activation of p38 MAPK (Figure 6B) and JNK (Figure 6D), but not of p42/44 MAPK (Figure 6C). As internal standards, the blots have been incubated with antibodies binding to the unphosphorylated forms of the kinases. Whereas JNK showed an early transient phosphorylation with a maximum at 1 h after stimulation (Figure 6D), the activation of p38 MAPK was demonstrated to occur in a biphasic manner with maximum phosphorylations at 1 and 4 h after IL-1β treatment (Figure 6B). The IL-1β-induced phosphorylation of both kinases was profoundly suppressed by WIN 55,212-2 following 1- and 4-h incubations with the test substances, respectively (Figure 6B, 6D). Incubation of HUVEC with WIN 55,212-2 alone did not affect the phosphorylation of JNK (Figure 6D) and p42/44 MAPK (Figure 6C). By contrast, an induction of p38 MAPK was observed 4 h after IL-1β stimulation, which was, however, not significant yielding phosphorylation levels below those observed in the IL-1β/WIN 55,212-2 group (Figure 6B).

**Figure 7:**
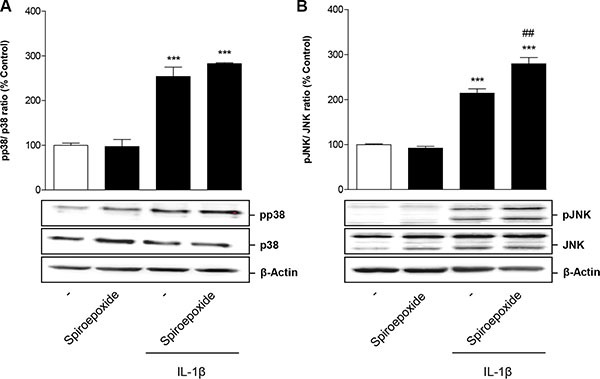
Impact of nSMase spiroepoxide inhibitor on IL-1β-induced activation of p38 MAPK (A) and JNK (B) Cells were pretreated with nSMase spiroepoxide inhibitor (10 μM) for 1.5 h. IL-1β or vehicle were added subsequently and the incubation was continued for 1 h. Percent control represents comparison with vehicle-treated cells (100%) in the absence of test substance. Values represent densitometric analyses and are means + SEM of *n* = 3 blots per group with the phosphorylated forms being normalized to the respective unphosphorylated form. ß-actin is shown as loading control. ***P < 0.001 vs. corresponding vehicle control; ^##^*P* < 0.01 vs. IL-1β, ANOVA plus post hoc Bonferroni test.

To investigate whether IL-1β-mediated p38 MAPK and JNK activation was a downstream event of nSMase-caused ceramide formation, cells were incubated with IL-1β in the presence of nSMase spiroepoxide inhibitor. However, analysis of both kinases showed no impact of the nSMase inhibitor on the phosphorylation of both p38 MAPK (Figure 7A) and JNK (Figure 7B).

### Impact of WIN 55,212-2 on IL-1β-induced TF expression in human monocytes

**Figure 8:**
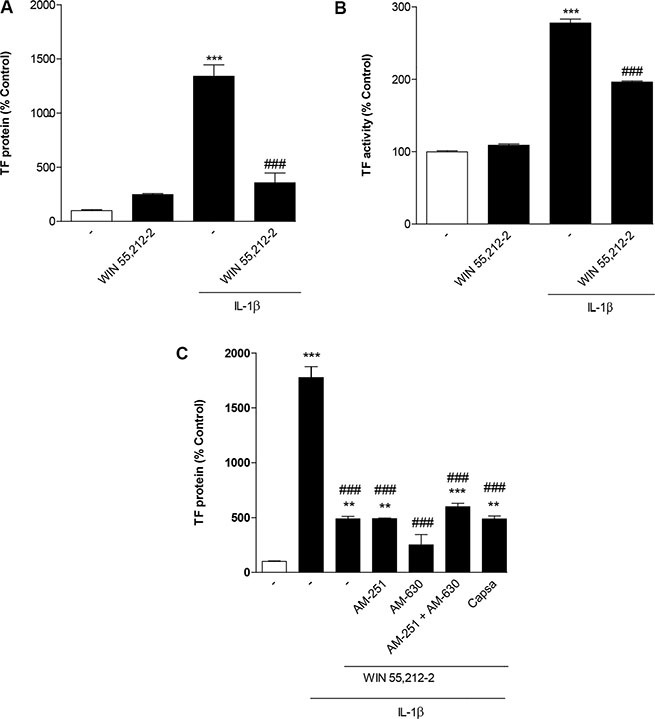
Impact of WIN 55,212-2 on basal and IL-1β-induced TF expression (A) and TF activity (B) in human blood monocytes Cells were incubated with WIN 55,212-2 at 10 μM for 8 h in the presence or absence of IL-1β. **(C)** Effect of AM-251 (CB_1_ antagonist), AM-630 (CB_2_ antagonist) and capsazepine (Capsa, TRPV1 antagonist) on the WIN 55,212-2-mediated inhibition of IL-1β-induced TF protein expression. Cells were pretreated with the respective receptor antagonist (all tested at a final concentration of 1 μM) for 1 h. IL-1β, WIN 55,212-2 (10 μM) or vehicle were added subsequently and the incubation was continued for 8 h. Percent control represents comparison with vehicle-treated cells (100%) in the absence of test substance. Values are means + SEM of *n* = 3 per group. **P < 0.01, ***P < 0.001 vs. corresponding vehicle control; ^###^*P* < 0.001 vs. IL-1β, ANOVA plus post hoc Bonferroni test.

To exclude that the observed inhibitory action of WIN 55,212-2 was restricted to HUVEC, additional key experiments were performed using human blood monocytes. In these cells, WIN 55,212-2 was likewise shown to interfere with IL-1β-induced TF protein expression (Figure 8A) and TF activity (Figure 8B). In addition, an increase of basal TF protein expression but not TF activity was observed (Figure 8A, 8B). Measurement of cellular viability by use of the WST-1 test revealed the following viability rates after an 8-h treatment with the respective substances: vehicle, 100% ± 2%; WIN 55,212-2 (10 μM), 80% ± 4% **; IL-1β, 102% ± 5%; IL-1β + WIN 55,212-2 (10 μM), 89% ± 4%; means ± SEM of *n* = 12 per group using monocytes from 2 different donors; **P < 0.01 vs. vehicle, one-way ANOVA plus post hoc Bonferroni test.

As shown for HUVEC before, the inhibitory effect of WIN 55,212-2 on IL-1β-induced TF expression was not reversed when monocytes were preincubated with antagonists to CB_1_, CB_2_ or TRPV1 (Figure 8C). Control experiments revealed no impact of the receptor antagonists on basal TF protein levels (data not shown) but a significant upregulation of IL-1β-induced TF in cells incubated with AM-630 as well as the combination of AM-630 with AM-251 (data not shown).

## DISCUSSION

**Figure 9:**
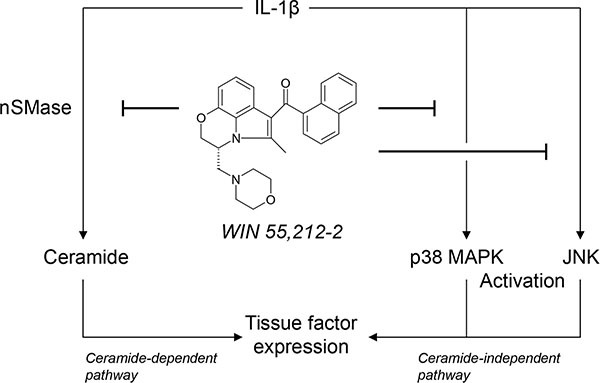
Scheme showing the proposed mechanism underlying the inhibitory effect of the synthetic cannabinoid WIN 55,212-2 on endothelial expression of tissue factor (TF) The proinflammatory cytokine interleukin (IL)-1β induces TF expression in endothelial cells by separate pathways involving ceramide formation or activation of p38 mitogen-activated protein kinase (MAPK) and c-Jun N-terminal kinases (JNK). WIN 55,212-2 inhibits IL-1β-induced TF expression via a receptor-independent pathway resulting in a suppression of neutral sphingomyelinase (nSMase)-dependent ceramide formation as well as an interference with the activation of p38 MAPK and JNK.

Although cannabinoids have emerged as potential therapeutic agents in diverse medical fields, their impact on hemostasis and thrombosis still remains controversial. The present study demonstrates the synthetic cannabinoid WIN 55,212-2 to decrease endothelial expression and activity of TF, a pivotal initial factor in blood coagulation and thrombus propagation. The mechanism elicited by WIN 55,212-2 was shown to include an interference with IL-1β-induced ceramide formation and activation of p38 MAPK and JNK (Figure 9).

Initial experiments demonstrated WIN 55,212-2 to cause a time- and concentration-dependent suppression of IL-1β-induced TF protein accompanied by decreases in TF mRNA and TF activity. WIN 55,212-2 showed a “threshold”-like profile with the inhibition of IL-1β-induced TF expression confined to micromolar concentrations. Inhibitor approaches addressing the role of cannabinoid receptors and TRPV1 revealed neither receptor to be involved in WIN 55,212-2-mediated inhibition of IL-1β-induced TF expression. Moreover, and in line with a cannabinoid receptor-independent event, the action of WIN-55,212-2 was mimicked by its cannabinoid receptor-inactive enantiomer WIN 55,212-3.

A receptor-independent inhibition of IL-1β-induced TF protein by WIN 55,212-2 was likewise demonstrated in human blood monocytes, suggesting that decreased expression of TF is part of a general anticoagulant mechanism of this cannabinoid. This view is also supported by a decrease of TF activity in IL-1β-stimulated monocytes exposed to WIN 55,212-2. Although the extent of TF protein induction in vascular cells has been reported to not always correlate with TF activity [43], the data presented here show a good correlation between both parameters in monocytic and endothelial cells.

In our hands WIN 55,212-2 only partially affected IL-1β-induced TF expression in HUVEC. This result is in line with other work showing only partial inhibition of endothelial TNF-induced TF expression by cardiac glycosides [44], dimethyl sulfoxide [45], celecoxib [46] or amiodarone [47]. Independent thereof, the functional relevance of the WIN 55,212-2-elicited inhibition of TF expression and activity was substantiated by data showing a significant reversal of the IL-1β-induced decrease of aPTT by the investigated cannabinoid. The aPTT represents a global test of endogenous coagulation that is primarily dependent on factors of the intrinsic system. However, its modulation by WIN 55,212-2 confirms a functional consequence of the cannabinoid-driven decrease of IL-1β-induced TF to likewise occur under conditions when both TF-driven extrinsic as well as TF-independent intrinsic coagulation system are activated.

With respect to the underlying mechanism, a decreased generation of cellular ceramide was found to confer one part of the inhibitory action of WIN 55,212-2 on IL-1β-induced TF expression in HUVEC. There are several lines of evidence supporting this notion. First, treatment of cells with nSMase spiroepoxide inhibitor [41], which selectively inhibits the nSMase-catalyzed hydrolysis of membrane sphingomyelin to ceramide, was associated with a substantial suppression of IL-1β-induced TF formation and cellular ceramide levels. This observation is in line with previous investigations showing activation of nSMase to be involved in signal transduction pathways elicited upon cellular activation with IL-1β [48–50]. Second, triggering the ceramide pathway by C_2_-ceramide, a cell-permeable ceramide analog, as well as by bacterial nSMase, which mimicked cellular nSMase activity, potentiated IL-1β-induced TF expression in an overadditive manner. Third and most importantly, IL-1β led to a significant increase of cellular ceramide levels with the resulting induction being fully reversed by WIN 55,212-2.

Although our study found a cannabinoid receptor-independent action of WIN 55,212-2, the data, however, present the first evidence for decreased ceramide formation by a cannabinoid compound. Previous investigations addressing the impact of cannabinoids on ceramide reported increased levels of this lipid second messenger resulting in several biological responses including induction of tumor cell apoptosis [51, 52], effects on energy metabolism [53], stimulation of ketogenesis [54] and glucose metabolism [55]. Concerning the impact of sphingolipids on TF, hitherto published data imply a cell- and substance-dependent action. Accordingly, sphingosine-1-phosphate but not C_2_-ceramide was shown to potentiate thrombin-induced TF expression in endothelial cells without itself inducing TF expression [56]. In other investigations, TF activity was upregulated by C_2_-ceramide in HUVEC [57] and by C_8_-ceramide or SMase in astrocytes [58]. On the other hand, inhibition of SMase by small molecules or siRNA conferred an upregulation of TF expression in human vascular smooth muscle cells via a mechanism involving membrane sphingomyelin enrichment [59]. Finally, an inhibitory action on hemostasis has been proposed for sphingosine that may suppress monocyte TF-initiated coagulation by inhibiting factor VII binding to TF [60].

During past years several studies have indicated p38 [43, 61, 62] and p42/44 MAPKs [43, 61] as well as JNK [43, 62, 63] as upstream regulators of TF expression. On the basis of inhibitor approaches showing a partial inhibition of IL-1β-induced TF expression by p38 MAPK and JNK inhibitors as well as Western blots indicating an activation of these kinases by IL-1β, both enzymes were identified as additional triggers of IL-1β-elicited TF expression. Subsequent experiments revealed WIN 55,212-2 to impair the phosphorylation of p38 MAPK and JNK thereby proving an additional target of the cannabinoid in interfering with TF formation by IL-1β.

As a matter of fact, p38 MAPK and JNK may be activated in a ceramide-dependent manner. Accordingly, both kinases have been shown to confer the ceramide-induced expression of matrix metalloproteinase-1 [64] and COX-2 [65]. However, in our hands the phosphorylation of either of these kinases was not suppressed by the nSMase spiroepoxide inhibitor indicating IL-1β-mediated p38 MAPK and JNK activation to occur independent of nSMase-caused ceramide formation. The existence of two separate pathways conferring IL-1β-induced endothelial TF expression (i.e., a ceramide-dependent signalling and a ceramide-independent response involving activation of MAPK) was further substantiated by the observation that inhibitors of both pathways (nSMase spiroepoxide inhibitor, SB203580, SP600125) led to partial inhibitions of IL-1β-induced TF expression only. Moreover, the importance of both pathways is underlined by the fact that treatment of HUVEC with WIN 55,212-2 without concomitant IL-1β stimulation was associated with a profound increase of cellular ceramide but no significant phosphorylation of p38 MAPK as well as JNK eventually resulting in an increase of TF protein below that observed after treatment with IL-1β. In further agreement with this notion, incubation of cells with C_2_-ceramide or nSMase from *B. cereus* without concomitant treatment with IL-1β elicited measurable but not significant inductions of TF expression in the time period studied.

Concerning the activation of p38 MAPK and JNK by IL-1β that seems to occur separately from the IL-1β-triggered nSMase/ceramide pathway, our data raise the question how ceramide may stimulate TF expression independent of these kinases. There are several mechanisms that appear feasible. First, a possible target would be the tyrosine kinase Scr that has recently been reported to be involved in endothelial TF expression [66]. In glial cells for instance, IL-1β was shown to mediate IL-6 secretion via a mechanism involving nSMase2-mediated Src activation [67] without activating p42/44 MAPK, JNK and p38 MAPK. Second, a ceramide-dependent modulation of other pathways conferring modulation of endothelial TF expression such as nuclear factor (NF)-kappa B [68] or PI3K/Akt [66, 69] appears likely. Third, a previous study indicated TNF to generate at least two endothelial cell inflammatory responses comprising a ceramide-dependent Raf-1 and p42/44 MAPK activation and a ceramide-independent efficient NF-kappa B translocation and activation of p38 and JNK-1 MAPK [70]. Noteworthy, our experiments revealed no induction of p42/44 MAPK by IL-1β, although PD98059, an inhibitor of p42/44 MAPK activation, caused a partial, but significant suppression of IL-1β-induced TF protein levels. Although we cannot exclude an IL-1β/ceramide-triggered activation of p42/44 MAPK at earlier time points than those studied, the data obtained with PD98059 should be interpreted with caution. Accordingly, a recently published investigation reported PD98059 to exert off-target effects independent of its ability to inhibit p42/44 MAPK activation [71].

There are some issues that merit special reference. First, in view of the receptor-independent action of WIN 55,212-2, it was surprising for us that this action was not mimicked by other cannabinoids. However, a comparable specificity for WIN 55,212-2 in exerting receptor-independent cannabinoid actions has been likewise observed by others [32, 72]. For example, WIN 55,212-2 has been shown to inhibit the TNF-induced neutrophil transmigration across human endothelial cells [32], whereas the cannabinoid agonists HU-210, CP 55,940 and AEA were inactive in this respect. However, in our hands, the inhibitory effect of WIN 55,212-2 on IL-1β-induced TF expression was unique for the aminoalkylindole and was not shared by diverse structurally different cannabinoids including endocannabinoids/endocannabinoid derivatives bearing an arachidonate structure (AEA, MA, 2-AG), phytocannabinoids with terpenoid structure bearing a closed (THC) or open (cannabidiol) pyrane ring or a synthetic cannabinoid with terpenoid structure (JWH-133). Among these compounds a substantial upregulation of IL-1β-induced TF protein was noticed for MA, AEA and JWH-133. A closer analysis of MA indicated a time-dependent upregulation of basal and IL-1β-induced TF protein levels with both effects occurring in a cannabinoid receptor and TRPV1-independent manner. Noteworthy, the potentiation of IL-1β-induced TF expression by the arachidonic acid derivative MA was not mimicked by arachidonic acid and was not inhibited by the COX inhibitor indometacin, thus excluding de novo synthesized COX products to mediate this response. In fact, IL-1β and MA have been shown to elicit an overadditive induction of COX-2 expression in neuroglioma cells [73]. In addition, a COX-2-mediated pathway for AEA metabolism has been demonstrated previously [74]. Independent thereof, more research is clearly needed to understand the actual, obviously diverse targets of WIN 55,212-2 within the IL-1β-induced endothelial TF expression.

Second, incubation of HUVEC with WIN 55,212-2 was associated with a significant decrease of basal viability. However, the possibility that decreased availability of TF by WIN 55,212-2 in IL-1β-treated HUVEC was an unspecific cytotoxicity-related phenomenon can be excluded due to the fact that all presented TF values had been normalized to cellular protein and β-actin, respectively. Decreased viability rates of HUVEC after incubation with WIN 55,212-2 have been reported before, even at lower nanomolar concentrations [75]. Although cytotoxic properties of WIN 55,212-2 may limit its therapeutic value referring to cardiovascular disorders, direct inhibition of vascular endothelial survival has been defined as one mechanism conferring anti-angiogenic properties of cannabinoids in context with their anti-cancer action [75]. Thus, in view of studies reporting TF expression by endothelial cells to enhance angiogenesis [5] and TF to be expressed on endothelial cells within the tumor vasculature [4–9], it is tempting to speculate that inhibition of endothelial TF expression by WIN 55,212-2 along with a moderate cytotoxic action may contribute to the anti-angiogenic properties reported for this cannabinoid [75, 76]. However, more research including analyses of TF expression in tumor cells is needed to understand the functional consequence of decreased TF generation by WIN 55,212-2 in context with tumor progression.

In summary, this study provides first-time proof for an inhibitory action of the synthetic cannabinoid WIN 55,212-2 on IL-1β-induced TF expression. The receptor-independent mechanism was shown to involve suppression of cellular ceramide and impaired activation of p38 MAPK and JNK. In view of the fact that increased TF action has been associated with diverse pathophysiologic disorders, further studies addressing the hitherto unknown anticoagulatory impact of this compound are recommended.

## MATERIALS AND METHODS

### Materials

WIN 55,212-2, JWH-133, cannabidiol, MA and PD98059 were purchased from R&D Systems, Inc. (Wiesbaden-Nordenstadt, Germany). WIN 55,212-3 was from LGC Standards (Wesel, Germany). 2-AG was obtained from Cayman Chemical (Ann Arbor; Michigan, USA). AM-251, AM-630, AEA, arachidonic acid, SB203580 and SP600125 were bought from Enzo Life Sciences (Lörrach, Germany). Capsazepine, indometacin, myriocin from *Mycelia sterilia* (ISP-1), C_2_-ceramide (N-acetyl-D-sphingosine), dihydro-C_2_-ceramide (D-erythro-N-acetylsphinganine) and nSMase from *Bacillus cereus* were obtained from Sigma-Aldrich (Taufkirchen, Germany). IL-1β, penicillin-streptomycin and trypsin/EDTA were from Invitrogen Life Technologies (Darmstadt, Germany). THC and nSMase spiroepoxide inhibitor were from Lipomed (Weil am Rhein, Germany) and Santa Cruz Biotechnology (Heidelberg, Germany), respectively. Fumonisine B_1_ from *Fusarium monoliforme* was from Merck Millipore (Darmstadt, Germany). Fetal calf serum (FCS) was obtained from PAN Biotech GmbH (Aidenbach, Germany).

### Cell culture

HUVEC, basal endothelial cell growth medium and supplements (endothelial cell growth supplement/heparin [ECGS/H], FCS, epidermal growth factor [EGF], basic fibroblast growth factor [bFGF], hydrocortisone) were obtained from PromoCell GmbH (Heidelberg, Germany). For all experiments HUVEC derived from the same umbilical cord (Caucasian, female genotype) were used at passage 4. HUVEC were maintained in basal endothelial cell growth medium containing supplements (0.4% ECGS/H [final concentration of heparin in medium: 90 μg/ml], 2% FCS, 0.1 ng/ml EGF, 1 ng/ml bFGF, 1 μg/ml hydrocortisone), 100 U/ml penicillin and 100 μg/ml streptomycin. Likewise, all incubations were performed using this medium. The cells were grown in a humidified incubator at 37°C and 5% CO_2_.

Fumonisine B_1,_ IL-1β, indometacin and nSMase were dissolved in aqua ad iniectabilia and diluted with PBS. 2-AG, AEA, arachidonic acid, cannabidiol, dihydro-C_2_-ceramide, JWH-133, MA, THC and WIN 55,212-2 were dissolved in ethanol and diluted with PBS to yield final concentrations of 0.1% (v/v) ethanol. AM-251, AM-630, capsazepine, C_2_-ceramide, nSMase spiroepoxide inhibitor, PD98059, SB203580, SP600125, WIN 55,212-3 were dissolved in DMSO and diluted with PBS to yield final concentrations of 0.1% (v/v) DMSO. In case of combined administration of two antagonists (AM-251, AM-630) the final concentration of DMSO was likewise limited to 0.1% (v/v) DMSO. ISP-1 was dissolved in methanol and diluted with PBS to yield final concentrations of 0.1% (v/v) methanol. As vehicle control PBS containing the respective amount of ethanol, DMSO or methanol was used. When necessary multiple vehicle solutions were added.

### Isolation and culture of mononuclear cells

Buffy coats obtained from human blood donations of healthy donors were kindly provided by Prof. V. Kiefel (Institute of Transfusion Medicine, Rostock University Medical Center). Buffy coats were diluted up to 1:4 with Dulbecco's phosphate-buffered saline (DPBS; PAN Biotech GmbH). 20 ml of lymphocyte separation medium LSM 1077 (PAA Laboratories GmbH, Pasching, Austria) was carefully overlaid with 30 ml of the diluted buffy coat mixture and centrifuged at 1200 × g for 20 min at room temperature. Centrifugation was performed without brake at acceleration and deceleration in order to obtain a phase separation into 4 layers: a pellet of erythrocytes and granulocytes at the bottom followed by a Ficoll phase, an interphase containing peripheral blood mononuclear cells (PBMC) and an outer plasma phase with thrombocytes. The plasma phase was removed to harvest the PBMC of the interphase properly. The collected PBMC were washed twice in warm DPBS and pelleted after each washing step by centrifugation at 300 × g for 10 min at room temperature. PBMC were seeded at a density of 11 to 12 million cells/well in 6-well plates and were incubated for 1 h in RPMI 1640 medium with 300 mg/l (2.1 mM) glutamine (Lonza, Cologne, Germany), 40 U/ml penicillin, 40 μgl/ml streptomycin (Invitrogen Life Technologies) and 10% heat-inactivated FCS in a humidified incubator at 37°C and 5% CO_2_. Within this time monocytes became adherent, whereas lymphocytes remained in the supernatant. Supernatants were removed and the cell layer was carefully washed twice with warm DPBS and subsequently stimulated with the respective test compounds or vehicles for the indicated time periods. All incubations of monocytes were performed in serum-free RPMI 1640 medium containing glutamine and antibiotics.

### Determination of TF protein

HUVEC were seeded at a density of 400,000 cells/well in 6-well plates 24 h before stimulation. PBMC were seeded at a density of 11 to 12 million cells/well in 6-well plates followed by a 1-h adhesion period and removal of supernatants. HUVEC or adherent monocytes were incubated with the respective test compounds or vehicles for the indicated time periods. Thereafter, supernatants were removed and cells were lysed in Cell Lysis Buffer 1 (R&D Systems) for 45 min under shaking conditions (1400 rpm, 25°C). Afterwards, lysates were centrifuged at 14,000 g for 5 min at 4°C. Supernatants were used for measurement of TF protein that was quantified using the Quantikine^®^ Human Coagulation Factor III/Tissue Factor Immunoassay (R&D Systems) according to the manufacturer's instructions. TF protein was normalized to total protein amounts determined in the respective supernatant using the bicinchoninic acid assay (Pierce, Rockford, IL, USA).

### Determination of TF mRNA

HUVEC were seeded at a density of 100,000 cells/well in 24-well plates 24 h before stimulation. After incubation of cells with the respective test compounds or vehicles for the indicated time periods, supernatants were removed and cells were lysed for subsequent RNA isolation. Total RNA was isolated using the RNeasy total RNA Kit (Qiagen, Hilden, Germany). β-Actin- (internal standard) and TF mRNA levels were determined by quantitative real-time RT-PCR as described previously [77]. Primers and probes for human β-actin and TF were TaqMan Gene Expression Assays (Applied Biosystems, Darmstadt, Germany).

### Determination of TF activity

HUVEC were seeded at a density of 400,000 cells per well in 6-well plates 24 h before stimulation. PBMC were seeded at a density of 11 to 12 million cells/well in 6-well plates followed by a 1-h adhesion period and removal of supernatants. HUVEC or adherent monocytes were incubated with the respective test compounds or vehicles for the indicated time periods. Thereafter, supernatants of HUVEC or monocytes were removed and cells were lysed under shaking conditions (1400 rpm, 15 min, 37°C) by use of the membrane detergent n-octyl-β-D-glucopyranoside (15 mM). Afterwards, lysates were centrifuged at 14,000 g for 5 min at 4°C. The obtained supernatants were used for measurement of TF protein activity with the Human TF Chromogenic Activity Assay Kit (Two step, Apo) (Gentaur, Aachen, Germany) according to the manufacturer's instructions. Briefly, this assay measures the amidolytic activity of the complex factor VIIa/ TF to cleave factor X directly to factor Xa which in turn perpetuates the cleavage of the chromogenic substrate to the chromophore para-nitroaniline (pNA) resulting in a yellow color change detectable at 405 nm. The grade of extinction of pNA correlates directly with the enzymatic TF activity. TF activity levels were normalized to cellular protein.

### Determination of activated partial thromboplastin time (aPTT)

HUVEC were seeded at a density of 400,000 cells per well in 6-well plates 24 h before stimulation. Following the desired incubation time with the respective test compounds or vehicles, supernatants were removed and cells were lysed under shaking conditions (1400 rpm, 15 min, 37°C) by use of the membrane detergent n-octyl-β-D-glucopyranoside (15 mM). Afterwards, lysates were centrifuged at 14,000 g for 5 min at 4°C. The obtained supernatants were used for measurement of aPTT. For this purpose pooled citrated plasma from patients (*n*= 5–10) of mixed gender without hemorrhagic diathesis was used. Plasma was kindly provided by the Institute of Clinical Chemistry, Rostock University Medical Center. For aPTT measurement 300 μl citrated pooled plasma was mixed with 100 μl supernatant on ice in 2.5 ml AXSYM sample cups and read out with the BCS^®^ XP coagulation analyzer (Siemens AG, Munich, Germany). Supernatants as well as pooled plasma probes were subjected to maximal one freeze-thaw cycle.

### Western blot analysis

HUVEC were seeded at a density of 400,000 cells/well in 6-well plates 24 h before stimulation and incubated with test substances or vehicle for the indicated time periods. Afterwards, HUVEC were lysed in RIPA buffer (0.05 mM Tris pH 8.0, 150 mM sodium chloride, 1% (v/v) IGEPAL CA-630, 5 mg/ml sodium desoxycholate, 1 mg/ml sodium dodecyl sulfate), incubated for 30 min while shaking at 4°C and centrifuged at 14,000 g for 5 min at 4°C thereafter. Supernatants were used for Western blot analysis. Total protein in the supernatant was measured using the bicinchoninic acid assay (Pierce). Proteins were separated on a 10% sodium dodecyl sulfate-polyacrylamide gel. After transfer to nitrocellulose membrane, blots were probed with specific antibodies raised to p38 MAPK, phospho-p38 (Thr180/Tyr182) MAPK, p42/44 MAPK, phospho-p42/44 (Thr202/Tyr204) MAPK, JNK, phospho-JNK (Thr183/Tyr185) SAPK (New England BioLabs GmbH, Frankfurt, Germany) or with a monoclonal anti-β-actin antibody (Sigma-Aldrich). Subsequently, membranes were probed with horseradish peroxidase-conjugated anti-rabbit IgG for p38 MAPK, phospho-p38 MAPK, p42/44 MAPK, phospho-p42/44 MAPK, JNK, phospho-JNK SAPK or horseradish peroxidase-conjugated anti-mouse IgG for β-actin (New England BioLabs GmbH). Antibody binding was visualized by a chemiluminiferous solution (100 mM Tris-HCl pH 8.5, 1.25 mM luminol, 200 μM p-coumaric acid, 0.09% [v/v] H_2_O_2_, 0.0072% [v/v] DMSO). Densitometric analysis of band intensities was achieved by optical scanning and quantifying using the Quantity One 1-D Analysis Software (Bio-Rad, Munich, Germany).

### Determination of ceramide levels

#### Materials

Pure standard of endogenous ceramide (C_16_) and HPLC-grade solvents for ESI–MS and lipid extraction were purchased from Sigma-Aldrich. The non-naturally occurring internal standard C_17_-ceramide (N-heptadecanoyl-D-erythro-sphingosine, d18:1/17:0; purity > 99%) was obtained from Avanti Polar Lipids (Alabaster, Alabama, USA).

#### Sample preparation for ceramide analysis

Cell sample pellets were resuspended in 1 ml of 20 mM Tris buffer (pH 8.0), spiked with 50 ng/ml of C_17_-ceramide and lysed using a Sonopols U-tip sonifier (Bandelin, Berlin, Germany). The lysates were then transferred to ice-cold screw-capped glass tubes. In parallel with standard solutions, samples were extracted using a protocol slightly modified to that published by Bligh and Dyer [78] with 1 ml of chloroform/methanol (1:2) and additionally 2 × 1 ml chloroform. The pooled organic phase was dried under nitrogen, and the residues were reconstituted in 100 μl acetonitrile/2-propanol (60:40, v/v) containing 0.2% formic acid for analysis.

#### LC-MS analysis

Extracted samples (60 μl) were analysed on a Waters HPLC 2695 Separation Module using a Multospher 120 C18 column 125 × 2 mm, 5-μm particle size (CS-Chromatographie Service GmbH, Langerwehe, Germany) coupled with a guard column (20 × 2 mm, 5-μm particle size). The ceramides were resolved using a gradient starting from 6% mobile phase A (water containing 0.2% formic acid) at a flow rate of 0.15 ml/min to 100% mobile phase B (acetonitrile/2-propanol [60:40, v/v] containing 0.2% formic acid) over 15 min at a linear gradient, and then with 100% phase B for 5 min. The column was then reequilibrated for 5 min with 96% mobile phase B. The HPLC column effluent was introduced into a Micromass Quattro Micro^TM^ API mass spectrometer (Waters, Milford, MA, USA) and analyzed using electrospray ionization in the positive mode and a single ion monitoring (SIM) modus: *m/z* 520.5 for C_16_-ceramide, *m/z* 575.5 for the C_17_-ceramide internal standard. The mass spectrometer and source parameters were set up as described previously [79]: capillary voltage, 3.0 kV; cone voltage, 40 V; source temperature, 120°C; desolvation temperature, 250°C, flow rate of desolvation gas, 700 l/h. Dwell and delay times were 0.1 and 0.05 s, respectively. The data were acquired using MassLynx software (version 4.1, Micromass Ltd, Manchester, UK). Ceramide levels were normalized to cellular protein.

#### Determination of cellular viability

HUVEC were seeded at a density of 25,000 cells/well in 96-well plates 24 h before stimulation. PBMC were seeded at a density of 100,000 cells/well in 96-well plates followed by a 1-h adhesion period and removal of supernatants. HUVEC or adherent monocytes were incubated with the respective test compounds or vehicles for 8 h in 100 μl of the corresponding medium. Thereafter, cell viability was measured by the colorimetric WST-1 test (Roche Diagnostics, Mannheim, Germany). This test is based on the cleavage of the tetrazolium salt 4-[3-(4-Iodophenyl)-2-(4-nitrophenyl)-2*H*-5-tetrazolio]-1,3-benzene disulfonate (WST-1) by mitochondrial dehydrogenases in metabolically active cells.

#### Statistics

Comparisons between groups were performed with one-way ANOVA plus post hoc Bonferroni (comparison of selected groups within one experiment) or Dunnett test (comparison of all groups versus vehicle) using GraphPad Prism 5.04 (GraphPad Software, Inc., San Diego, USA). Results were considered to be statistically significant at values of *P* < 0.05.

## Abbreviations

AEA: N-arachidonoylethanolamine (anandamide)
2-AG: 2-arachidonoylglycerol
AM-251: N-(piperidin-1-yl)-5-(4-iodophenyl)-1-(2,4-dichlorophenyl)-4-methyl-1 H-pyrazole-3-carboxamide
AM-630: (6-iodo-2-methyl-1- [2-(4-morpholinyl)ethyl]-1H-indol-3-yl) (4-methoxyphenyl) methanone
CB1: cannabinoid receptor 1
CB2: cannabinoid receptor 2
IL-1β: interleukin-1β
JWH-133: (6aR,10aR)-3-(1,1-Dimethylbutyl)-6a,7,10,10a-tetrahydro-6,6,9-trimethyl-6H-dibenzo[b, d]pyran
MA: R(+)-methanandamide
MAPK: mitogen-activated protein kinase
nSMase: neutral sphingomyelinase
nSMase spiroepoxide inhibitor: N-[(2R)-3-hydroxy-1-oxo-1-[(4-oxo-1-oxaspiro[2.5]octa-5,7-dien-7-yl)amino]propan-2-yl]decanamide
PD98059: 2-(2-amino-3-methoxyphenyl)-4H-1-benzopyran-4-one
RT-PCR: reverse transcriptase-polymerase chain reaction
SB203580: 4-(4-fluorophenyl)-2-(4-methylsulfinylphenyl)-5-(4-pyridyl)idazole, SP600125, Anthra(1,9-cd)pyrazol-6(2H)-one
THC: Δ9-tetrahydrocannabinol
TRPV1: transient receptor potential vanilloid 1
WIN 55,212-2: (R)-(+)-[2,3-Dihydro-5-methyl-3-(4-morpholinylmethyl)pyrrolo[1,2,3-de]-1,4-benzoxazin-6-yl]-1-naphthalenylmethanone mesylate
WIN 55,212-3: (3S)-2,3-Dihydro-5-methyl-3-(4-morpholinylmethyl)pyrrolo[1,2,3-de]-1,4-benzoxazin-6-yl]-1-naphthalenyl-methanone monomethanesulfonate
WST-1: 4-[-(4-iodophenyl)-2-(4-nitrophenyl)-2H-5-tetrazolio]-1,3-benzene disulfonate.
